# Epid‐based in vivo dose verification for lung stereotactic treatments delivered with multiple breath‐hold segmented volumetric modulated arc therapy

**DOI:** 10.1002/acm2.12538

**Published:** 2019-02-20

**Authors:** Savino Cilla, Anna Ianiro, Maurizio Craus, Pietro Viola, Francesco Deodato, Gabriella Macchia, Milly Buwenge, Alessio G. Morganti, Vincenzo Valentini, Angelo Piermattei

**Affiliations:** ^1^ Medical Physics Unit Fondazione di Ricerca e Cura Giovanni Paolo II ‐ Università Cattolica del Sacro Cuore Campobasso Italy; ^2^ Radiation Oncology Unit Fondazione di Ricerca e Cura Giovanni Paolo II ‐ Università Cattolica del Sacro Cuore Campobasso Italy; ^3^ Radiation Oncology Department DIMES Università di Bologna ‐ Ospedale S.Orsola Malpighi Bologna Italy; ^4^ Radiation Oncology Department Fondazione Policlinico Universitario A. Gemelli ‐ Università Cattolica del Sacro Cuore Roma Italy; ^5^ Medical Physics Unit Fondazione Policlinico Universitario A. Gemelli ‐ Università Cattolica del Sacro Cuore Roma Italy

**Keywords:** in‐vivo dosimetry, quality assurance, SBRT, VMAT

## Abstract

We evaluated an EPID‐based in‐vivo dosimetry (IVD) method for the dose verification and the treatment reproducibility of lung SBRT‐VMAT treatments in clinical routine. Ten patients with lung metastases treated with Elekta VMAT technique were enrolled. All patients were irradiated in five consecutive fractions, with total doses of 50 Gy. Set‐up was carried out with the Elekta stereotactic body frame. Eight patients were simulated and treated using the Active Breath Control (ABC) system, a spirometer enabling patients to maintain a breath‐hold at a predetermined lung volume. Two patients were simulated and treated in free‐breathing using an abdominal compressor. IVD was performed using the SOFTDISO software. IVD tests were evaluated by means of (a) ratio R between daily in‐vivo isocenter dose and planned dose and (b) *γ*‐analysis between EPID integral portal images in terms of percentage of points with *γ*‐value smaller than one (*γ*_%_) and mean *γ*‐values (*γ*_mean_) using a 3%(global)/3 mm criteria. Alert criteria of ±5% for R ratio, *γ*_%_ < 90%, and *γ*_mean_ > 0.67 were chosen. 50 transit EPID images were acquired. For the patients treated with ABC spirometer, the results reported a high level of accuracy in dose delivery with 100% of tests within ±5%. The *γ*‐analysis showed a mean value of *γ*_mean_ equal to 0.21 (range: 0.04–0.56) and a mean *γ*_%_ equal to 96.9 (range: 78–100). Relevant discrepancies were observed only for the two patients treated without ABC, mainly due to a blurring dose effect due to residual respiratory motion. Our method provided a fast and accurate procedure in clinical routine for verifying delivered dose as well as for detecting errors.

## 1 INTRODUCTION

The technological advancements in immobilization and imaging, together with the ability to deliver high conformal doses and to account for organ motion have led to a widespread implementation of stereotactic body radiotherapy (SBRT) in a number of clinical settings.^1^ Over the last few years, different new techniques including volumetric modulated arc therapy (VMAT) have been successfully applied to SBRT treatments, owing to high‐dose conformity, improved sparing of healthy tissues and fast delivery time.^2^, ^3^, ^4^, ^5^ In SBRT treatments effective tumor motion control must be considered much more compelling because reduced margins are needed to avoid the irradiation of a large amount of normal tissue to high doses. Various methods have been developed to explicitly account for respiration motion in SBRT treatments, as respiratory gating techniques, breath‐hold techniques, and forced shallow breathing techniques.^6^ In particular, Active Breath Control (ABC) methods by means of spirometers have been used to actively hold the patient's breath at a certain level (e.g., at moderate deep inhalation) during the beam‐on time, providing an accurate method to improve the localization of lung and liver tumors during SBRT delivery.^7^, ^8^ This strategy has been recently implemented for SBRT treatments of extracranial metastases, with the aim to increase the accuracy of treatment delivery and the intrafraction treatment reproducibility.

The integration of several complex techniques such as SBRT, VMAT, and breath‐hold into a single therapeutic strategy requires a high‐level of confidence in the accuracy of the entire treatment delivery process. The impact of treatment delivery errors represents a major concern for these complex techniques, suggesting a strong argument in favor of in‐vivo dosimetry (IVD).^9^ In particular, due to very large dose over a few number of fractions used in SBRT treatments, any error in treatment delivery can be much more detrimental as compared to conventional fractionated therapy, with a major risk of completely nullify the curative intent or produce serious damage to the patient. Among the different available dosimetric systems, amorphous silicon electronic portal imaging devices (aSi‐EPID) have demonstrated unique favorable characteristics for IVD purposes (high two‐dimensional resolution and fast image acquisition) and several algorithms were developed to reconstruct the dose within the patient in terms of point dose, 2D‐dose distribution or 3D dose distribution.^10^

At our institution, we developed a generalized procedure for the daily in‐vivo isocenter dose reconstruction of radiation treatments,^11^, ^12^, ^13^, ^14^, ^15^ leading to the foundation of an Italian national project financed by the Istituto Nazionale di Fisica Nucleare^16^ and to the development of the SOFTDISO software (Best Medical Italy, Italy). The analysed results for conformal radiotherapy reported clinically relevant differences between planned and delivered dose, detecting the presence of dose discrepancies in more than 10% of the tests with respect to our tolerance levels.^17^ Recent publications have investigated the new challenges of epid‐based IVD for more complex treatments as IMRT or VMAT, showing the feasibility and the sensitivity of this approach to detect dose discrepancies also for these complex techniques.^18^, ^19^, ^20^ By routine clinical use of EPID‐based IVD, major dosimetric discrepancies due to anatomical variations^21^ and serious errors including plan transfer errors due to record‐and‐verify network failure were detected.^22^ A large clinical experience has been recently carried out at the Radiotherapy Centre of the Fondazione Policlinico Universitario A. Gemelli in Rome by means of an automated Epid‐based IVD procedure for more than 800 patients. The application of IVD procedure has allowed the authors to detect on average 6% of VMAT plans and 21% of 3D‐CRT plans outside at least one of their tolerance levels.^23^

Experiences with Epid‐based IVD for SBRT treatments are even more rare, with very few publications showing the technical feasibility of this strategy. McCowan et al. have recently validated an in‐house physics‐based model which utilizes EPID images to reconstruct the dose in patient during SBRT‐VMAT treatments.^24^ The authors reported satisfactory results for lung and spine cases, with pass rates better than 93% with 3%‐3 mm *γ*‐index tolerance level, showing the suitability of this approach for clinical implementation. Recently, a review by McCurdy and McCowan^25^ presented the technical aspects of in vivo dosimetry for lung SBRT, with a special emphasis on Epid‐based IVD as a powerful tool to identify errors that would have been missed with other common quality assurance systems. The application of in vivo dosimetry to lung SBRT presents new challenges with respect to other anatomical sites. These were mainly due to the presence of low density tissues surrounding the tumor, then straining the scatter estimation due to the loss of electronic equilibrium, and to the accuracy of the treatment planning system algorithm to properly calculate the patient dose. Two recent studies^26^, ^27^ have investigated the effectiveness of epid‐based IVD systems in detecting deliberately introduced errors during VMAT. Although both studies had an exiguous number of patients in the SBRT setting, they showed the ability of epid‐based IVD to detect serious errors in dose delivery, anatomical variations of patients or wrong set‐up.

We have recently applied our IVD algorithm, widely used for 3D‐conformal, IMRT and VMAT, to lung SBRT treatments, with the aim to supply, in quasi real‐time, both the isocenter dose and the *γ*‐analysis of transit EPID images. In this paper, we presented our current experience on real patients with EPID‐based IVD for the dose verification of lung SBRT treatments delivered with breath‐hold multisegmented VMAT technique.

## 2 MATERIAL AND METHODS

### 2.A Simulation, active breath control and treatment planning

Ten patients with lung metastases treated with Elekta VMAT were enrolled. Patients set‐up was carried out with the stereotactic body frame (SBF, Elekta, Crawley, UK), an immobilization device used to define target position by a stereotactic coordinates system instead of anatomical landmarks or skin markers,^28^ with an attached “vacuum pillow” customized to each patient. For eight of the ten enrolled patients, the breathing control was performed using the ABC system (Elekta, Crawley, UK), a spirometer able to immobilize the respiratory motion repeatedly and reproducibly for a period of time that can be comfortably tolerated by the patient.^7^, ^8^

Before final CT acquisition for planning, patients underwent a 3‐day training to assess their comfort and compliance, the lung capacity and the optimal breath hold length/level.

At the end, all patients underwent a moderate deep inspiration breath‐hold at 75% to 80% of maximum inspiration capacity, and a breath‐hold length of 20–30 s. During this training, three CT scan studies were acquired in order to evaluate the interfraction reproducibility of tumor position.

Two patients were not compliant to perform deep inspiration breath‐hold using the ABC system due to their small lung tidal volume. For these two patients a forced shallow breathing was performed by means of an abdominal plate compressor, with the aim to reduce the diaphragmatic excursions while still permitting limited normal respiration.

The clinical target volume was defined as the gross tumor volume. The planning target volume (PTV) was individually defined for each patient based on Internal Margin (IM) and Setup Margin (SM) assessment. The IM was defined based on the residual respiratory excursions; the SM margin was set at 3 mm for all patients.

Dose prescription was 50 Gy in five fractions for all patients. VMAT plans were generated with Ergo++ treatment planning system (TPS) (Elekta, Crawley, England). This is an anatomy‐based TPS that supplies a simplified approach to create VMAT plans by manually predefining a series of aperture shapes in conjunction with the beam's eye view (BEV) of the target and organs at risk. Planning procedure was reported in details in a previous study.^4^ However, because the delivery of a SBRT‐VMAT arc takes more than a single tolerable breath‐hold, we designed a solution to perform a full arc rotation delivery splitting the arc into short sub‐arcs, for each of which the delivery time was defined according to the patient predefined breath‐hold period. In the first step, the aperture shape for each control point within a sub‐arc was determined by the BEV of the target and the adjacent critical structures, automatically adapting the leaf edge to the outline of the PTV. Then, the beam weights for all the control points were optimized by inverse planning based on the simulated annealing optimization algorithm, so defining the dose‐rate/monitor units number ratio for each control point. All plans were optimized with a single full arc. The entire gantry rotation was described in the optimization process by a sequence of 90 control points, i.e., one every 4°.

Patient set‐up was checked before every treatment fraction using the portal images obtained by two perpendicular square open 10 × 10 cm^2^ beams (each of which delivers two monitor units) and their comparison with the corresponding digitally reconstructed radiographs obtained from the planning CT dataset. Any deviation greater than 3 mm in the isocenter position was immediately corrected. The EPID portal image obtained at the end of the delivery of the VMAT arc in the first treatment fraction was assumed as the reference image for the subsequent daily *γ*‐analysis. In other words, the first portal image was used as a surrogate of EPID transit signals to ensure the highest treatment reproducibility. This means that once the patient setup is corrected before each treatment fraction, IVD *γ*‐analysis should supply correct results if there are no linac or no breath‐control system failures.

### 2.B The SOFTDISO software

SOFTDISO is a commercial system for IVD developed within an Italian National research project.^16^ The mathematical aspects of the dose reconstruction algorithm were deeply explained in a previous paper.^20^ The SOFTDISO software is directly interfaced with the Record & Verify system of the radiotherapy network (Mosaiq, Elekta, Crawley, UK) and consists of two integrated modules. The first one, called the “Patient commissioning module”, imports the DICOM files from the CT scanner and the TPS in order to extract all the needed radiological and geometrical parameters. The second module, called the “Test Computation Module”, was developed to obtain the R ratio between the daily reconstructed dose at isocenter and the planned isocenter dose. The accuracy of this procedure has been well reported in literature^19^ providing a tolerance level of ±5%. The module supplies also the *γ*‐analysis for daily EPID images with respect to a reference one. The *γ*‐analysis tests were evaluated in terms of percentage of points with *γ* value less than one (*γ*_%_) and in terms of mean *γ* values (*γ*_mean_). Based on previous experience on other anatomical sites,^20^, ^21^ a 3% (global)‐3 mm criteria was adopted. Alert criteria of *γ*_%_ < 90% and *γ*_mean_ > 0.67 were chosen, to accept only 10% of values above 3%/3 mm and an average discrepancy of 2%/2 mm. These criteria seems to be a reasonable choice to provide the detection of significant errors but without a major number of false positives.

The EPID portal images were automatically imported by the SOFTDISO software via DICOM protocol and the IVD results were delivered to a computer screen in quasi real‐time, that is within one minute from the end of the arc delivery.

### 2.C IVD clinical workflow

Electronic portal imaging devices‐based IVD was performed for each treatment fraction for all enrolled patients, with the aim to steadily track the treatment reproducibility (mainly, in terms of setup inaccuracy and/or breath‐hold failure). If the R ratio and/or the *γ*‐analysis values should exceed the tolerance levels, then the medical physicist examines the IVD chain in order to detect and remove any possible sources of errors. When the dose discrepancies were unclear, a new CT scan was required for the patient resimulation and replanning. At the end of the treatment, as the final act of a multistep quality assurance process,^29^ an IVD report is inserted into the patient's medical chart, providing a record of the actual dose received by individual patients.

## 3 RESULTS

**Figure 1:**
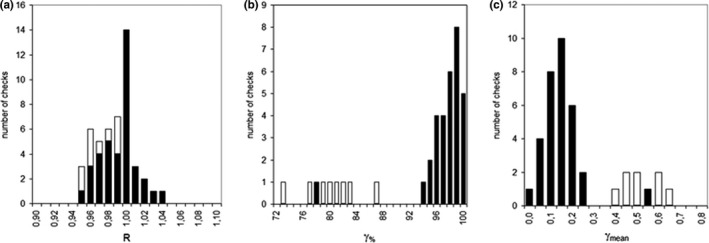
Distribution of (a) R ratios, (b) *γ*_%,_ and (c) *γ*_mean_ values for the 10 patients. Black bars indicate the tests for the patients treated with the ABC system; white bars indicate the tests for the two patients treated in free‐breathing.

A total of 50 transit EPID images, one image for each SBRT‐VMAT plan, was acquired during the treatment fractions of the 10 patients. Two images were removed from analysis for an electronic acquisition failure. Figure 1 shows the R ratios, *γ*_%_ and *γ*_mean_ histogram values for all patients; the black bars refer to the eight patients treated with ABC and the white bars to the two patients treated in free‐breathing. Table 1 reports the overall results for R, *γ*_%_ and *γ*_mean_ metrics.

**Table 1:** Overall results for R, *γ*_%_ and *γ*_mean_ metrics

	Patients treated with ABC (n = 8)			Patients treated without ABC (n = 2)		
	R ratio	*γ*_%_	*γ*_mean_	R ratio	*γ*_%_	*γ*_mean_
Mean values	0.993	96.9	0.21	0.972	80.1	0.52
Range	0.95–1.04	78–100	0.04–0.56	0.95–0.99	73–87	0.38–0.66
% within tolerance level	100	98	100	100	0	100

**Figure 2:**
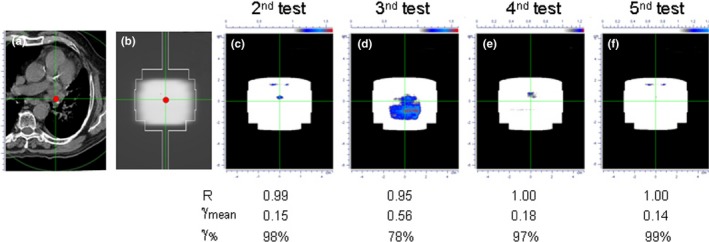
Detection of a poor treatment reproducibility in a patient treated using the ABC spirometer. From left to right, (a) the CT scan at isocenter level; (b) the reference integrated EPID image; (c–f) the IVD tests with a relevant discrepancy provided by *γ*‐analysis in figure (d).

For the eight patients treated with ABC spirometer, the results show a very high level of accuracy in dose delivery, with no dose discrepancies in any patient (except one test) and a very high interfraction reproducibility. In particular, only one fraction of a patient of this cohort supplied one of the three metrics out of tolerance level, with *γ*_%_ value equal to 78% (while R ratio and *γ*_mean_ values were equal to 0.95 and 0.56, respectively). Figure 2 shows the detection of this discrepancy, due a slight variations in the tidal volume of the patient above the threshold value, leading to a small displacement of the lesion. Excluding from the analysis the only test providing the aforementioned dose discrepancy, the mean R ratio resulted equal to 0.994 (range: 0.961–1.042), with 100% of tests within ±5%. The results for *γ*‐analysis showed an average values of *γ*_%_ and *γ*_mean_ equal to 97.5 (range: 94%–100%) and 0.19 (range: 0.04–0.29), respectively, with 100% of tests within alert criteria for both metrics.

**Figure 3:**
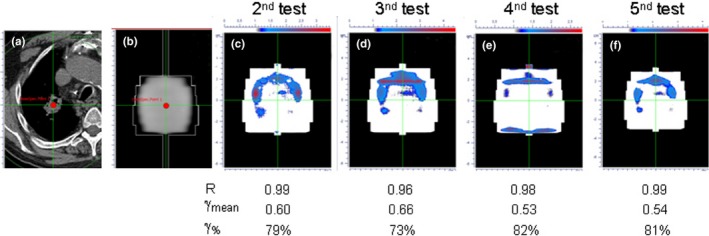
Detection of dose discrepancies in a patient treated in free‐breathing using IVD procedure. From left to right, (a) the CT scan at isocenter level; (b) the reference integrated EPID image; (c–f) the IVD tests showing a poor treatment reproducibility probably due to the residual tumor motion and a blurring dose effect.

Larger discrepancies were observed only for the two patients treated without the ABC, and where all described by the *γ*_%_ tests outside the alert criteria. In fact, for these two patients the R ratios were in the range of 0.95–0.99 for all the tests, that is, no dose discrepancies outside tolerance level were observed at the isocenter point. Similarly, the *γ*_mean_ tests provided values smaller than 0.67 (range: 0.38–0.66). However, the 2D *γ*‐analysis clearly reported large off‐axis deviations in *γ*_%_, which dropped below 90% for all the tests. Figure 3 shows an example of poor treatment reproducibility for one of these patients; this graphical result could be justified by a dose blurring effect due to the residual respiration motion of the lesion across the beam central axis, mainly in the cranio‐caudal direction.

## 4 DISCUSSION

Stereotactic body radiotherapy has become a critical component of the cancer treatment process, demonstrating a great efficacy in many disease sites. This complex and technology‐driven clinical procedure requires the highest level of attention for safe application. In the last years, serious errors have occurred leading to severe patients’ complications, including a linac calibration error in Florida (affecting 77 patients in 2004–2005), wrong measurements of output factors both in France (affecting 145 patients in 2006–2007) and in Missouri (affecting 152 patients in 2004–2009,^30^ and the well‐known lethal over‐irradiation of a patient in Scotland.^31^ A review of the Nuclear Regulatory Commission Radiation Event Report Notification database reported other 13 stereotactic‐related events in the period 2004–2010, mainly due to wrong targeting and isocenter setting and to errors in dose planning.^32^

The integration in SBRT process of advanced techniques as VMAT and gating/breath‐hold systems for organ‐motion control has the potential to further increase the accuracy and outcomes of SBRT treatment. On the other hand, an even higher level of confidence of the entire treatment delivery process is required, because each new implemented technique can in turn become a new source of error. For example, an error in respiratory management parameters for a patient receiving a SBRT lung treatment was documented, in which the gating phase and breathing cycle parameters were inadvertently changed during the course of the treatment.^33^ Following the occurrence of the aforementioned incidents, a number of guidance documents for the implementation and routine maintenance of high‐quality stereotactic programs have been published.^34^, ^35^ All these reports suggested the implementation of IVD in clinical routine, as the last hedge able to intercept machine and patient‐specific errors during the dose delivery. This suggestion should be mandatory when new advanced technologies and treatments are implemented in clinical routine and potential limitations still have to be assessed. In this case, since unexpected and unknown errors may occur, a robust IVD program should be able to trace deviations between planned and actually delivered doses that could be missed by pretreatment verification.^9^

We reported our initial experience for clinical application of IVD for complex SBRT‐VMAT treatments of lung metastases using a breath‐hold system for organ motion control. At present, our method supplies the daily dose reconstruction only along the beam axis at isocenter point, providing a robust warning for major dose variations (i.e., linac or other technical failures). Treatment reproducibility, instead, is performed by *γ*‐analysis comparison between daily portal images. We demonstrated that this procedure, even if it is not a true 2D dosimetric comparison, can trace back the causes of transit signal changes, allowing a prompt action to remove any clinically relevant dose discrepancies before compromising the aim of patient curative treatment.^21^ The small number of patients enrolled in the present study does not allow a statistically significant comparison between the results obtained for the patients treated with and without ABC system. However, this was not the aim of this paper that was instead focused on the ability of our IVD procedure to identify treatment‐related dose discrepancies also during SBRT‐VMAT delivery. This aim has been well‐illustrated by two examples. First, major discrepancies in treatment reproducibility were found for the two patients treated without ABC. In particular, we want to highlight that the observed discrepancies for these two patients were detected only by *γ*‐analysis of the 2D portal images. This finding was mainly due to the fact that in SBRT‐VMAT treatments of lung metastases, the isocenter is always located at the center of the lesion so that the beam central axis is always crossed by the tumor during the residual respiratory motion, preventing a detectable reduction of the radiological path and then relevant isocenter dose discrepancies. It must however be emphasized that, in the present clinical setting, the isocenter point is always located in the center of the tumor, in a homogenous dose region, thus representing a relevant point for dose verification purposes and providing, for example, a major warning for technical failure (linac output variations or MLC failures). In other anatomical sites as head‐neck or breast cancer, the isocenter point is often located in regions with high‐gradient dose or in very low dose regions, producing unreliable dose assessments. As shown in Fig. 3, the lack of reproducibility of the treatment is mainly located at the periphery of the lesion, suggesting a probable dose blurring effect due to the residual tumor motion. The impact of these discrepancies for the target volume remains difficult to quantify. In our anatomy‐based VMAT optimization strategy, the field shape always conform to the PTV outline, and this choice should avoid both target missing and possible interplay effect. Only a DVH‐analysis based on 3D IVD would allow a quantitative assessment of the clinical relevance of observed deviations.^36^, ^37^ Secondly, as shown in Fig. 2, a patient treated with ABC failed to fulfill the *γ*_%_ criterion, with a fall of *γ*_%_ down to 78%. Although still within tolerance levels, this test supplied also the worst result for the R ratio (R = 0.95) and for the *γ*_mean_ metric (*γ*_mean_ = 0.56). A careful inspection of possible causes highlighted small variations in held volume above the threshold value that may result in a variation of the tumor position. This effect is probably due to the delay between the ABC activation and the balloon valve closing, depending on the flow rate when the threshold volume is reached, with high flow rates causing an overshoot of the threshold value.

Our back‐projection algorithm for IVD was developed by means of correlation functions between isocenter dose and EPID signals that were obtained using homogeneous phantoms.^10^, ^13^, ^18^ Although our model takes into account the amount of scatter radiation due to the distance of the patient from the Epid, it does not directly model the scatter contribution coming from low density tissues surrounding the tumor. Anyway, the goodness of our results for the isocenter dose reconstruction, reporting an agreement with planned dose within 5% in all tests, suggests that the loss of electronic equilibrium, which should result in an effectively tumor lower dose, can be considered negligible for the purposes of IVD in this clinical setting. In an our previous study^38^ we used an heterogeneous phantom to verify the agreement between the doses calculated with pencil beam algorithms, the in‐vivo dose and the doses measured along the beam central axis (with a small ionization chamber), which crossed a target in water equivalent material surrounded by cork equivalent to typical lung density. For dynamic conformal arc technique the results reported an agreement within 2% (with a tendency to an overestimation of our algorithm). This result was obtained if the conformal arc irradiation of the lung target included a thickness of lateral low density cork medium less than 1 cm. This condition, typical in the setting of lung SBRT where margins around the target are less than 1 cm, assured that the effect of the lateral disequilibrium is negligible for the in vivo dose reconstruction at isocenter (e.g., the center of the lung lesion).

It must be underlined that all the detected dose discrepancies would not have been detected by means of pretreatment dose verification. In our clinical workflow for SBRT quality assurance, pretreatment dosimetric verification is performed for all plans using a stringent local 3%‐3 mm *γ* criterion with the aim to detect any sources of errors related to treatment planning system and/or to the ability of linac to deliver the planned dose. Only plans resulting in a *γ*% pass‐rate higher than 95% are accepted. Then, epid‐based IVD is performed to daily monitor any errors that may occur during the treatment fractions. A global 3%‐3 mm *γ* criterion was chosen for the IVD tests to avoid an excess of high false‐positive events that could frequently discontinue the treatment to trace‐back small dose discrepancies without any clinical impact. This way, the main goal of our IVD approach is to efficiently detect the dose discrepancies that are potentially clinically significant, rather than achieve the highest accuracy in the dose reconstruction. The choice of three simultaneous pass/fail criteria should reduce the risk of missing major clinically relevant dose discrepancies.

In particular, our overall results confirm the correctness of the adopted tolerance/action levels of 5% for R ratios, and >90% of *γ*_%_ pass‐rate and <0.67 for *γ*_mean_ when using a 3%(global)/3 mm criteria. For the eight patients that underwent ABC treatment, 100% of the R ratio, *γ*_mean_ and *γ*_%_ tests resulted within their respective alert criteria, excluding one test for which the reasons for the disagreement have been understood. The adopted tolerance levels clearly demonstrated the power to highlight even small discrepancies in the treatment reproducibility. In particular, the *γ*_%_ pass‐rate metric resulted in the best discriminator for the detection of discrepancies in the treatment reproducibility. However, it must be underlined that all tests for patients treated with the ABC system provided *γ*_mean_ values less than 0.33 (excluding the aforementioned single test showing dose discrepancy). This means that the reproducibility of our breath‐hold SBRT‐VMAT treatments could be tested at a level of average discrepancies of 1%/1 mm, suggesting the use of lower criteria for *γ*_mean_ in this clinical setting (e.g., *γ*_mean_<0.33). In other words, the average *γ*_mean_ value of 0.19 (range: 0.04–0.29) found in this clinical setting means that the average dose differences among the various treatment fractions is less than 1%, indicating a very high treatment reproducibility for patients treated with the ABC system, and allowing the use of the stringent *γ*_mean_<0.33 criterion. The values for the alert criteria used in the clinical application for the different *γ*‐evaluation indicators deserve further deeper investigations. The recent recommendations of the AAPM Task Group No. 218^39^ highlighted that discrepancy tolerance limits are neither well‐defined nor consistently applied across Centers in patient‐specific IMRT quality assurance. In general, the definition of tolerance and action levels is much more challenging for in vivo dosimetry purposes, mainly depending on the ability of the IVD equipment to detect errors requiring corrections. In particular, the dose delivery process performance outside action levels should be considered unacceptable and demanding actions to promptly correct. Following our past experience,^21^ the action levels were set equal to the tolerance levels, so as to investigate any test outside the tolerance levels and to trace back possible errors in (a) linac or other equipment failure, (b) patient set‐up, (c) breath‐hold control device failure, and (d) patient's morphological changes. We are currently performing a comprehensive sensitivity analysis to ensure the best detectability for several types of errors in the various clinical settings, including SBRT.

An ideal IVD system should produce results instantaneously, during the beam delivery, in order to detect gross error as they occur, e.g., before the delivery of the whole dose to the patient. In the past years, we explored this feasibility for dynamic conformal arc therapy.^38^ Our aim was to monitor the treatment reproducibility in real time using a transmitted current signal sampled at interval time of 110 ms supplied by a small ionization chamber located at portal level. This strategy was then successfully applied to monitor the tumor position reproducibility during the treatment of lung cancer patients treated with 3D‐conformal therapy and using the ABC spirometer.^40^ However, the transition to an Epid‐based IVD for online treatment verification poses serious technological issues still today that prevent its manageable clinical implementation. Our previous research^41^ reported the feasibility to obtain the “real‐time” isocenter dose in dynamic conformal arc therapy using the signal supplied by an EPID cine acquisition mode every 1.66 s during the treatment. A more complex experience of real‐time IVD using Epids was reported by Woodruff et al.,^42^ who demonstrated the feasibility of this approach in a clinical setting for head‐neck and pelvic IMRT and VMAT treatments. The authors developed a real‐time IVD method based on the comparison between measured and predicted portal images, showing the feasibility to catch large delivery errors. A research on real‐time Epid‐based 3D dose reconstruction, also using DVH‐analysis, is ongoing at the Netherland Cancer Institute (NKI), where the authors have successfully combined their online dose verification system with a linac halting mechanism in cases of major discrepancies.^43^ Obviously, a true real‐time IVD approach is even more stringent for single fraction radiosurgical treatments.

## 5 CONLUSIONS

In conclusion, our preliminary results suggested that our IVD method provide a fast and accurate procedure in clinical routine for verifying delivered dose as well as for detecting dose discrepancies in lung SBRT treatments. IVD has been shown to identify dose discrepancies that would have been missed with other quality assurance methods. The use of ABC in lung SBRT translates in very high reproducibility treatments.

## CONFLICT OF INTERESTS

We certify that any actual or potential conflicts of interest do not exist regarding this paper; the work is original, has not been accepted for publication, nor is concurrently under consideration elsewhere, and that all the authors have contributed directly to the planning, execution, or analysis of the work reported or to the writing of the paper.
